# Impact of self-reported fasting duration on lipid profile variability, cardiovascular risk stratification and metabolic syndrome diagnosis

**DOI:** 10.20945/2359-3997000000023

**Published:** 2018-03-23

**Authors:** Carolina Castro Porto Silva Janovsky, Antonio Laurinavicius, Fernando Cesena, Viviane Valente, Carlos Eduardo Ferreira, Cristovão Mangueira, Raquel Conceição, Raul D. Santos, Marcio Sommer Bittencourt

**Affiliations:** 1 Universidade Federal de São Paulo Universidade Federal de São Paulo São Paulo SP Brasil Endocrinologia e Metabolismo, Universidade Federal de São Paulo (Unifesp); Medicina Preventiva, Hospital Israelita Albert Einstein (HIAE), São Paulo, SP, Brasil; Hospital Israelita Albert Einstein Hospital Israelita Albert Einstein São Paulo SP Brasil; 2 Hospital Israelita Albert Einstein Hospital Israelita Albert Einstein São Paulo SP Brasil Medicina Preventiva, Hospital Israelita Albert Einstein (HIAE), São Paulo, SP, Brasil; 3 Hospital Israelita Albert Einstein Hospital Israelita Albert Einstein Laboratório Clínico São Paulo SP Brasil Laboratório Clínico, Hospital Israelita Albert Einstein (HIAE), São Paulo, SP, Brasil; 4 Hospital Israelita Albert Einstein Hospital Israelita Albert Einstein São Paulo SP Brasil Medicina Preventiva, Hospital Israelita Albert Einstein (HIAE); Instituto do Coração, Hospital das Clínicas, Faculdade de Medicina da Universidade de São Paulo (InCor-HCFMUSP), São Paulo, SP, Brasil; Universidade de São Paulo Universidade de São Paulo Faculdade de Medicina Hospital das Clínicas São Paulo SP Brasil; 5 Hospital Israelita Albert Einstein Hospital Israelita Albert Einstein Faculdade de Medicina São Paulo SP Brasil Faculdade de Medicina – Medicina Preventiva, Hospital Israelita Albert Einstein (HIAE), São Paulo, SP, Brasil

**Keywords:** Triglycerides, cardiovascular risk, metabolic syndrome, fasting

## Abstract

**Objective:**

We sought to investigate the impact of self-reported fasting duration times on the lipid profile results and its impact on the cardiovascular risk stratification and metabolic syndrome diagnosis.

**Subjects and methods:**

We analyzed data from all consecutive individuals evaluated in a comprehensive health examination at the Hospital Israelita Albert Einstein from January to December 2015. We divided these patients in three groups, according to the fasting duration recalled (< 8h, 8-12h and > 12h). We calculated the global cardiovascular risk and diagnosed metabolic syndrome according to the current criteria and estimated their change according to fasting duration.

**Results:**

A total of 12,196 (42.3 ± 9.2 years-old, 30.2% females) patients were evaluated. The distribution of cardiovascular risk was not different among groups defined by fasting duration in both men and women (p = 0.547 for women and p = 0.329 for men). Similarly, the prevalence of metabolic syndrome was not influenced by the fasting duration (p = 0.431 for women and p = 0.166 for men).

**Conclusion:**

Self-reported fasting duration had no significant impact on the lipid profile results, including triglyceride levels. Consequently, no changes on the cardiovascular risk stratification using the Framingham risk score nor changes on the prevalence of metabolic syndrome were noted.

## INTRODUCTION

Current guidelines on screening for cardiovascular risk rely on the measurement of plasma cholesterol levels as part of the initial risk stratification (1–3). Those guidelines also recommend that the lipid panel measurement should be performed after an 8- to 12-hour fast. This fasting request is proposed as a way to eliminate any interference of the postprandial lipid levels, particularly for triglycerides, and to allow the use of previously validated cut-offs for diagnosis and management of dyslipidemia (3,4). However, this long fasting period may be cumbersome and may lead to important logistic difficulties to patients.

Interestingly, prior evidence suggests that the lipid profile variability is relatively small in healthy individuals (5,6). Other groups have already analyzed the interference of fasting on the lipid profile, and their results suggest that less than 12-hour fasting may offer important logistic advantages with minimal impact on the lipid panel results or cardiovascular risk stratification (7–9).

Despite those previous results on the lipid profile variability, few studies (10,11) have evaluated the impact of the fasting duration on cardiovascular risk stratification or metabolic syndrome diagnosis. Such information is necessary as the clinical relevance of the lipid profile variability mainly depends on its effects on cardiovascular risk stratification and, hence, on the clinical decision-making process. Therefore, in the present study we sought to investigate the impact of self-reported fasting duration time on the lipid profile results and its impact on the cardiovascular risk stratification and metabolic syndrome diagnosis.

## SUBJECTS AND METHODS

We selected all consecutive individuals evaluated at the Hospital Israelita Albert Einstein from January to December 2015. This evaluation was part of an executive checkup program paid by the employers from their organizations. The protocol includes extensive clinical and laboratory evaluations.

The Ethical Committee of the Hospital Israelita Albert Einstein approved this study and the consent form was waived as the research is based on database analysis and no additional patient contact was needed.

Participants were questioned about previous presence of dyslipidemia, systemic arterial hypertension (previous diagnosis, use of anti-hypertensive medicines or measured blood pressure ≥ 140/90 mmHg), diabetes (previous use of medicines for diabetes or fasting glycemia > 126 mg/dL) and smoking (consumption of at least one cigarette per day within the last 30 days). Body mass index (BMI) was measured using the weight/height^2^ (kg/m^2^) formula. The abdominal circumference was measured by a trained nutritionist and was considered abnormal when above 94 cm for men and 80 cm for women.

We included patients between 20 and 80 years of age and excluded those taking statins or with liver problems.

When the patients made the appointment for the comprehensive health examination, they were oriented to fast for at least 12 hours. On the day of the exam, they were asked about how long they have been fasting. Independently of the fasting time, all exams were collected. Therefore, the patients were analyzed considering the period of time they self-reported they were fasting. We divided these patients in three groups, according to self-reported fasting duration: less than 8 hours fasting, between 8 and 12 hours and more than 12 hours fasting.

We calculated the general cardiovascular risk, considering gender, age, systolic blood pressure, smoking, presence of diabetes, use of anti-hypertensive agents, HDL-cholesterol levels and total cholesterol levels. If the risk was lower than 10% in 10 years, it was considered low risk; between 10 and 20%, intermediate risk and higher than 20%, high risk for cardiovascular events in 10 years (12). This score calculates the risk of coronary death, myocardial infarction, coronary insufficiency, angina, ischaemic stroke, hemorrhagic stroke, transient ischemic attack, peripheral artery disease and heart failure in 10 years.

The diagnosis of metabolic syndrome was made if waist circumference was abnormal (≥ 94 cm in men and 80 cm in women) and at least two of these four elements were impaired: HDL-cholesterol, triglycerides, glycemia and blood pressure (13).

The measurement of lipoproteins (total cholesterol, HDL-C and triglycerides) was performed in automated equipment VITROS 5600^®^ Ortho Clinical Diagnostics by dry chemical colorimetric method. The LDL-C was calculated by Fridewald formula for triglycerides for concentrations up to 250 mg/dL. For values greater than 250 mg/dL, direct LDL-C was performed in automated equipment VITROS 5600^®^ Ortho Clinical Diagnostics by endpoint methodology. The LDL-C calculated loses correlation when compared to the gold standard that is ultracentrifugation. The higher the triglyceride value, the worst is this relationship (14). For this reason we use the calculation for values up to 250 mg/dL keeping the routine used by Albert Einstein Hospital laboratory.

### Statistical analysis

Continuous variables are presented as means and standard deviation or medians and quartiles, as appropriate, and compared using one-way ANOVA or Kruskal-Wallis test. Categorical variables are presented as absolute numbers and proportions, and compared using chi-square test.

In order to accommodate the significant difference in the distribution of gender across fasting duration, different cardiovascular risk calculators and differences in the definition of metabolic syndrome, we chose to perform a gender-stratified analysis across fasting duration groups.

Additionally, in order to adjust for the potential confounding effect of age, gender and waist circumference across the fasting duration groups, we chose to perform a multiple linear regression analysis on triglyceride levels.

A level of significance of 0.05 was used. All analysis were performed using Stata version 13.0 (StataCorp, USA).

## RESULTS

We included 12,196 patients that were divided in three groups according to the self-reported fasting time. The baseline characteristics of these groups are shown in Table 1. Due to the large sample size, almost all information was statistically different between the groups, though the absolute differences were small.

**Table 1:** Baseline characteristics stratified according to fasting duration

	Total (n = 12,196)	Less than 8 h fasting (n = 1,829)	Between 8 and 12 h fasting (n = 5,515)	More than 12 h fasting (n = 4,852)	p
Male	8,514 (69.8)	949 (52)	4,031 (73.1)	3,534 (72.8)	< 0.001
Age (y)	42.3 ± 9.2	41.4 ± 9.0	42.5 ± 9.0	42.2 ± 9.4	< 0.001
BMI (kg/m^2^)	26.3 ± 4.2	26.0 ± 4.5	26.2 ± 4.1	26.5 ± 4.2	< 0.001
Waist circumference (cm)	91 ± 15.0	89 ± 13.6	91 ± 12.5	92 ± 17.9	< 0.001
SBP (mmHg)	115.6 ± 12.0	114.6 ± 12.6	115.7 ± 12.0	115.9 ± 11.9	< 0.001
DBP (mmHg)	76.2 ± 8.2	76.4 ± 8.7	76.0 ± 8.1	76.3 ± 8.0	0.096
Total Cholesterol (mg/dL)	191.8 ± 34.4	193.6 ± 35.3	190.5 ± 34.5	192.7 ± 34.0	< 0.001
LDL-c (mg/dL)	118.5 ± 31.4	117.9 ± 32.3	118.1 ± 31.4	119.1 ± 31.1	0.236
HDL-c (mg/dL)	48.8 ± 14.0	51.8 ± 15.5	48.3 ± 13.6	48.2 ± 13.7	< 0.001
Tryglicerides (mg/dL)	124.3 ± 83.7	119.4 ± 82.4	121.6 ± 76.0	129.3 ± 83.7	< 0.001
Glucose (mg/dL)	87.0 ± 13.1	82.0 ± 12.6	87.9 ± 11.4	87.8 ± 14.5	< 0.001
HbA1c (%)	5.40 ± 0.5	5.37 ± 0.5	5.38 ± 0.5	5.42 ± 0.6	< 0.001
Diabetes	178 (1.5)	19 (1.0)	84 (1.6)	75 (1.6)	0.265
Hypertension	1331 (10.9)	175 (9.6)	607 (11.0)	549 (11.3)	0.119
Smoking	1049 (8.6)	175 (9.6)	447 (8.1)	427 (8.8)	0.029
Fasting duration (h)*	11.7 (10.2-12.4)	5.2 (4.0-6.2)	11.2 (10.3-11.6)	12.6 (12.2-13.2)	0.001

Values are mean ± SD or n (%).

* Median and quartiles.

BMI: body mass index; SBP: systolic blood pressure; DBP: diastolic blood pressure; LDL-c: low-density lipoprotein cholesterol; HDL-c: high-density lipoprotein cholesterol; HbA1c: glycated hemoglobin.

**Figure 1:**
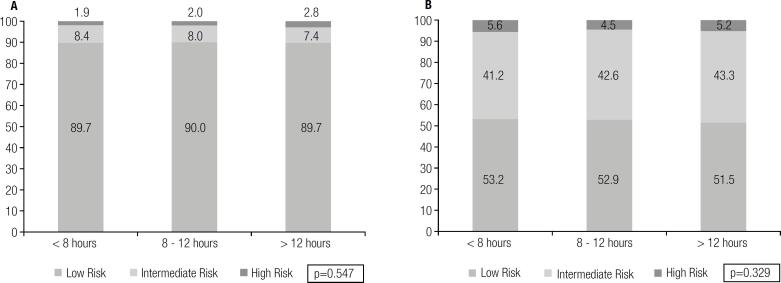
General cardiovascular risk according to fasting duration in women (**A**) and men (**B**).

**Figure 2:**
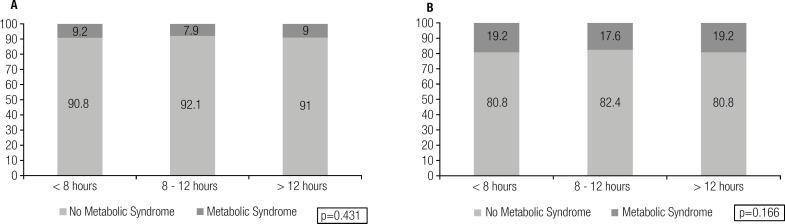
Metabolic syndrome prevalence according to fasting duration in women (**A**) and men (**B**).

For triglycerides, a small, albeit significant, increase was noted with longer fasting duration. However, after adjustment for the confounding effects of age, gender and waist circumference, no significant difference in triglyceride levels was observed (< 8 hours vs 8-12 hours: p = 0.114; < 8 hours vs > 12 hours: p = 0.220). Additionally, most risk factors were associated with triglyceride levels, though the median fasting duration was not different across triglycerides strata (Table 2). The small changes in the lipid profile across the fasting duration groups did not impact the overall distribution of cardiovascular risk irrespective of gender (Figure 1 – p = 0.547 for women and p = 0.329 for men). Similarly, the prevalence of metabolic syndrome was not influenced by the fasting duration in both genders (Figure 2 – p = 0.431 for women and p = 0.166 for men).

**Table 2:** Baseline characteristics stratified according to triglycerides levels

	Tg < 150 mg/dL (n = 9,131)	Tg 150 – 400 mg/dL (n = 2,927)	Tg > 400 mg/dL (n = 124)	p
Male	5,963 (65.31)	2,430 (83.02)	117 (94.35)	< 0.001
Age (y)	41.9 ± 9.3	43.4 ± 8.8	42.8 ± 8.2	< 0.001
BMI (kg/m^2^)	25.7 ± 4.0	28.2 ± 4.2	28.5 ± 3.8	< 0.001
Waist circumference (cm)	88.8 ± 15.4	97.5 ± 11.8	99.2 ± 10.1	< 0.001
SBP (mmHg)	114.2 ± 11.7	119.6 ± 12	123.6 ± 12.0	< 0.001
DBP (mmHg)	75.2 ± 8	79.1 ± 8	81.3 ± 8.2	< 0.001
Total Cholesterol (mg/dL)	185.5 ± 32	209.9 ± 34	232.2 ± 38.5	< 0.001
LDL-c (mg/dL)	115.8 ± 30.5	127.5 ± 32.1	100.6 ± 35.4	< 0.001
HDL-c (mg/dL)	51.5 ± 13.8	41.2 ± 11.1	31.3 ± 7.4	< 0.001
Glucose (mg/dL)	85.6 ± 10	90.7 ± 18.3	98.7 ± 30.3	< 0.001
HbA1c (%)	5.4 ± 0.4	5.5 ± 0.7	5.8 ± 1.2	< 0.001
Diabetes	90 (1.0)	79 (2.7)	9 (7.3)	< 0.001
Hypertension	826 (9.1)	481 (16.4)	23 (18.6)	< 0.001
Smoking	710 (7.8)	324 (11.1)	14 (11.3)	< 0.001
Fasting duration (h)*	11.7 (10.1-12.4)	11.8 (10.5-12.5)	11.5 (10-12.3)	< 0.0001

Values are mean ± SD or n (%).

* Median and quartiles.

BMI: body mass index; SBP: systolic blood pressure; DBP: diastolic blood pressure; LDL-c: low-density lipoprotein cholesterol; HDL-c: high-density lipoprotein cholesterol; HbA1c: glycated hemoglobin.

## DISCUSSION

We have shown that the self-reported fasting time had no significant impact on the lipid profile results. Consequently, no changes on the cardiovascular risk stratification using the Framingham risk score nor changes on the prevalence of metabolic syndrome were noted.

A recent review from the American College of Cardiology (15) has discussed the use of fasting or nonfasting samples depending on the question we need to answer. Although they do not provide extensive data to support the idea, they suggest that non-fasting samples are sufficient to estimate baseline cardiovascular risk in primary prevention and to define metabolic syndrome. Our findings provide robust real life data to support the expert opinion of this document, by addressing this issue in a large population of primary prevention adults undergoing a comprehensive heath examination.

Fasting has always been recommended prior to collecting serum lipid profile as the postprandial state is associated with a significant increase in triglycerides levels. As triglycerides are included in the Friedewald equation for calculating LDL-cholesterol levels, those changes in triglycerides levels could potentially affect the estimation of LDL-c and, therefore, impact the cardiovascular risk stratification. However, recent reports suggest that the impact of usual meals on triglyceride levels might be lower than previously estimated by fat tolerance tests. Thus, those reports agree that fasting prior to lipid profile evaluation might not be needed for most individuals (6,9,16).

Despite those recommendations, experts have proposed new cut-points for these exams when performed in non-fasting scenarios. A recent study from the Women's Health Study has determined a cut-off for triglycerides of 175 mg/dL when predicting cardiovascular risk using non-fasting samples (7). Similarly, the American College of Cardiology has established a threshold of 200 mg/dL for triglycerides when defining metabolic syndrome in a non-fasting state (15). Those recommendations contrast with the real life findings of our study, where the fasting duration did not significantly impact the lipid profile or the estimation of cardiovascular risk and metabolic syndrome based on it.

Since the need of an 8 to 12 hour fasting impacts on the logistic of laboratory centers, the recent recommendations, corroborated by our current findings may facilitate the organization of laboratory services and add comfort to patients. It would allow distributing the patients’ appointment throughout the day, avoiding long waiting hours in the morning, as well as increasing the overall laboratory capacity with the distribution of tests throughout the day.

Our study must, however, be read within the context of its design. First, we considered the fasting time recalled by the patients. It is probable that it was not exactly the same as the period that it really happened. However, it is difficult to find a way to measure this period without trusting the patient memory and all data on this subject considers the self-reported time. Not only that, patient recall of the last meal is the standard of care in real life. Second, the majority of our patients had low cardiovascular risk, which implies that the lipid profile is better and less likely to be affected by the fasting duration. Nonetheless, the main point of our findings is to improve patient's adherence, especially those testing for an initial lipid profile or for first global cardiovascular risk assessment in a check-up unit. Also, we didn't measure the lipid profile from the same patient with different fasting durations. Although it could generate some difference on the results, we understand that our population is homogeneous and the results from the group could be extrapolated to each individual. When analyzing the data, we could see that there were mild differences between the different fasting duration groups. It seems that older people with more comorbities (e.g. diabetes) are more likely to respect the fasting time, probably because they are more used to collect exams. Notwithstanding, these differences did not impact in our results and the guidance of non-fasting would be of great value for these patients in order to avoid hypoglycemia risk.

In conclusion, fasting does not impact the lipid profile testing in a comprehensive health examination, considering that the main purpose of this testing would be determining the global cardiovascular risk and metabolic syndrome diagnosis.
